# Reducing Malaria Transmission in Africa

**DOI:** 10.1371/journal.pmed.0020114

**Published:** 2005-04-26

There are 300 million cases of malaria each year worldwide, causing one million deaths. Around 90% of these deaths occur in Africa, mostly in young children. One of the greatest challenges facing Africa in the fight against malaria is drug resistance; resistance to chloroquine (CQ), the cheapest and most widely used antimalarial, is common throughout Africa, and resistance to sulfadoxine-pyrimethamine (SP), the first-developed and least expensive alternative to CQ, is also increasing in eastern and southern Africa. These trends have forced many countries to change their treatment policies and use more expensive drugs, including drug combinations that will hopefully slow the development of resistance. One avenue of research is to identify combinations that minimize gametocyte emergence in treated cases and prevent selective transmission of parasites resistant to any of the partner drugs.

In this month's *PLoS Medicine* Colin Sutherland and colleagues tested two leading combination therapies in children with uncomplicated malaria. One regimen was an artemisinin-based combination consisting of artemether and lumefantrine (co-artemether, trade names CoArtem and Riamet). The other was a combination of CQ and SP—currently under consideration in several African countries, largely due to its low cost. In this randomized, controlled trial, 497 children with acute uncomplicated falciparum malaria were given either a combination of CQ and SP or six doses of co-artemether (91 received CQ/SP and 406 received co-artemether), and their blood was tested for infectivity to mosquitoes seven days after treatment. During follow up at seven, 14, and 28 days the team found that children treated with co-artemether were significantly less likely to carry gametocytes in their blood than children treated with CQ and SP—7.9% compared with 48.8%.[Fig pmed-0020114-g001]

**Figure pmed-0020114-g001:**
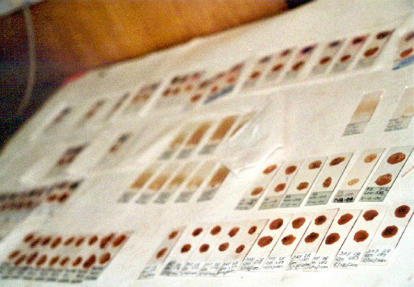
Thick blood films from patients in Gambia with malaria (Photo: Elisa Meier)

Altogether, the six-dose regimen of co-artemether was highly effective at reducing the prevalence and duration of gametocyte carriage. The numbers of gametocytes and the infectiousness to mosquitoes at day 7 were also reduced compared to a combination of CQ and SP, said the authors. Other studies have already shown the potential of co-artemether combination therapy to both cure malaria and reduce gametocyte carriage, acknowledged the authors. However, this study is the first to demonstrate the treatment's potential to markedly reduce the infectiousness of patients to mosquitoes, and has done so in a sub-Saharan African setting with highly seasonal transmission and where asymptomatic infections are common.

Do the results mean co-artemether should be introduced as a first-line treatment for malaria in Africa? The authors are hesitant and suggest there might be compliance issues with the six-dose regimen. The requirement of oily food for adequate absorption might also lead to inadequate drug levels in the blood of many treated individuals.

The authors suggest that co-artemether as a first-line treatment is not likely to reduce overall transmission of Plasmodium falciparum within the community but rather would reduce selective transmission of resistant parasites in treated patients. Hence, co-artemether could have a public health benefit by reducing the impact of drug resistance.

